# Renal biopsies from donors with acute kidney injury show different molecular patterns according to the post-transplant function

**DOI:** 10.1038/s41598-024-56277-x

**Published:** 2024-03-19

**Authors:** Flavia Neri, Maria Letizia Lo Faro, Maria Kaisar, Ka Ho Tam, Martyna Borak, Jan Lindeman, Annalisa Angelini, Marny Fedrigo, Jesper Kers, James Hunter, Rutger Ploeg

**Affiliations:** 1 Nuffield Department of Surgical Sciences, University of Oxford, Oxford, UK; 2 General Surgery 3 and Transplantation, Hospital Papa Giovanni XXIII, Square OMS 1, 24127 Bergamo, Italy; 3 Institute of Biomedical Engineering, University of Oxford, Oxford, UK; 4 Oxford Regional Genetics Laboratory, Oxford University Hospitals, Oxford, UK; 5 Department of Surgery, Leiden University Medical Center, Leiden, The Netherlands; 6 Pathology of cardiac transplantation and regenerative medicine unit Department of Cardiac, Thoracic and Vascular Sciences and Public Health, University of Padua, Padua, Italy; 7 Department of Pathology, Amsterdam UMC, University of Amsterdam, Amsterdam, The Netherlands; 8 Department of Pathology, Leiden Transplant Center, Leiden University Medical Center, Leiden, The Netherlands; 9 Van’t Hoff Institute for Molecular Sciences, University of Amsterdam, Amsterdam, The Netherlands

**Keywords:** Biomarkers, Outcomes research, Molecular biology, Biomarkers, Nephrology

## Abstract

The utilization of kidneys from donors with acute kidney injury (AKI) is often limited by unpredictable post-transplantation outcomes. The aim of our study was to identify protein mediators implicated in either recovery or failure of these organs. Forty kidney biopsies from donors with (20) and without AKI (20) were selected and then subdivided according to the post-transplant outcome defined as a threshold of 45 ml/min for the eGFR at 1 year from transplantation. Tissue homogenates were analysed by western blot to assess how the levels of 17 pre-selected proteins varied across the four groups. Samples from AKI kidneys with a poor outcome showed a fourfold increase in the levels of PPARg and twofold reduction of STAT1 compared to the other groups (p < 0.05). On the contrary, antioxidant enzymes including TRX1 and PRX3 were increased in the AKI kidneys with a good outcome (p < 0.05). An opposite trend was observed for the detoxifying enzyme GSTp which was significantly increased in the AKI group with poor versus good outcome (p < 0.05). The importance of lipid metabolism (PPARg) and inflammatory signals (STAT1) in the function recovery of these kidneys hints to the therapeutical targeting of the involved pathways in the setting of organ reconditioning.

## Introduction

Kidneys procured from donors with acute kidney injury (AKI) is still an underutilized resource due to clinical uncertainty and perception that AKI kidneys are associated with a higher risk of primary non-function and poor long-term outcomes, resulting in the decline or even discard of many suitable organs^1,2^. Recent studies have strengthened the concept of AKI and chronic kidney disease as two interconnected syndromes, with an increased risk of developing end-stage renal disease (ESRD) for those patients who survive an episode of AKI^3^. In the field of organ donation, AKI induces a dysregulation of metabolism and cellular stress repair mechanisms that may trigger chronic injury ultimately leading to earlier graft failure after transplantation^4^. In this study we have focused on the mechanisms of stress and metabolism involved in acutely injured kidneys that proceed towards transplantation.

The purpose of our study was to define and compare patterns of cellular and mitochondrial injury on outcomes after transplant in donor kidneys with and without acute injury before donation. We aimed to assess whether kidneys with acute injury prior to transplant had an imbalance between markers of regeneration and damage in the tissue at time of retrieval and whether that affected progression of renal function after transplantation.

## Materials and methods

### Study population

The QUOD biobank, started in 2013, is a national repository of samples collected from deceased donors throughout the United Kingdom according to predefined protocols and following appropriate informed consent by the donor families (https://quod.org.uk). All clinical samples are linked to corresponding donor and recipient demographic and clinical metadata, provided by the National Transplant Registry hosted by NHS Blood and Transplant.

Utilisation of QUOD samples for this study was granted under QUOD’s research ethics approval 18/NW/0187. All biopsies for this study were obtained from the upper pole of the kidney cortex at the back table preparation from donors deceased after either brain or circulatory death. Each kidney biopsy, performed using a 23 mm needle biopsy gun, was divided in two halves; one fixed in formalin and one stored in RNA-later and subsequently frozen in liquid nitrogen. All experiments were performed in accordance with national guidelines and regulations upon approval by the local ethical committee of QUOD project RAP017.

### Clinical variables

Acute kidney injury scoring of the donors was determined by calculating the fold change or difference between the levels of terminal creatinine (at the time of organ retrieval) and baseline creatinine (at the time of donor admission in intensive care); the parameter of urinary output was not considered as it was not always available for the donor. AKI was defined and graded in stages of severity according to the KDIGO guidelines^5^; mild (AKI 1) as an increase in the level of terminal creatinine of 0.3 mg/dl or more compared to the baseline, or a fold change rise of 1.5 to 1.9; moderate (AKI 2) as a fold change in the terminal creatinine between 2 and 2.9; severe (AKI 3) as a threefold rise in terminal creatinine or starting of renal replacement therapy. Clinical data included donor age, type of donation either donation after rain death (DBD) or donation after cardiocirculatory death (DCD), kidney donor risk index (KDRI) according to the calculator^6^, CIT (cold ischemia time), recipient age and BMI, sensitization status and pre-transplant dialysis duration.

A short-term indicator of transplantation outcome was necessary to distinguish between ‘poor’ and ‘good’ outcome. Previous studies have identified eGFR as a reliable indicator of transplant survival. Particularly, eGFR below 45 ml/min/1.73 mm at 12 month was observed to be a significant risk factor for graft failure^7^. Therefore, we adopted the same threshold to discriminate poor and good post-transplant outcome in our cohort of recipients.

### Selection of sample cohort

This study only considered deceased donors of kidneys that were successfully transplanted as single kidneys, excluding combined and dual kidney transplants. We also excluded cases where no adequate biopsy samples at retrieval were taken and no recipient eGFR at 1-year post-transplant was available. In this study we selected donors in whom kidney pairs had concordant outcomes in respect to 1 year eGFR. Donors whose kidneys yielded different outcomes after transplant in the corresponding recipients (i.e., one graft with ‘poor’ and the contralateral one with ‘good’ eGFR) were excluded to reduce the impact of recipient-related variation on outcomes.

The cohort of donors within the inclusion criteria was divided in groups according to the occurrence of AKI prior to retrieval or not. Finally, subgroups were defined according to the different post-transplant outcome as per the eGFR at 1 year: G1, donors with AKI and recipients with poor outcome (eGFR < 45 ml/min); G2, donors with AKI and recipients with good outcome (eGFR of ≥ 45 ml/min); G3, donors without AKI and recipients with poor outcome; G4, donors without AKI and recipients with good outcome.

In addition, we attempted to match donors to be included in this study from the pool of eligible QUOD donors (and thus the obtained tissue samples to be analysed) according to clinical variables: donor age, donor type, KDRI, cold ischaemia time, recipient age, recipient BMI, time on dialysis, number of re-transplantations. The selection of the donors allocated in the four groups also aimed to maximize the difference in eGFR at 12 months between the outcome groups.

### Western blot analysis

A panel of proteins selected for their potential involvement in AKI were analysed in the selected donor kidney biopsies by western blotting and included: peroxisome proliferator-activated receptor gamma coactivator 1-alpha (PGC1a), mitofusin 1 and 2 (MFN1 and MFN2), peroxisome proliferator-activated receptor gamma (PPARg), heat shock protein (Hsp70), hepatocyte growth factor (HGF), glutathione S-transferase alfa (GSTa), transforming growth factor beta (TGFb), thioredoxin (TRX), peroxiredoxin (PRDX3), signal transducer and activator of transcription (STAT1), dynamin-related protein (DRP1) and phospho-DRP1, platelet derived growth factor receptor (PDGFRa), motif chemokine receptor 1 (CX3CR1), gamma-glutamyltransferase (gGT), Insulin-like growth factor-binding protein (IGFBP), glutathione S-transferase (GSTp). Detailed methods can be found in the Supplementary Material.

### Histopathological assessment

The baseline donor chronic kidney injury and acute kidney injury were assessed by histological scoring using the formalin fixed, paraffin-embedded part of the core needle biopsy. Kidney paraffin sections (4 μm) were deparaffinized, rehydrated, and stained with Haematoxylin and Eosin (Leica Infinity H&E stain), according to manufacturer’s instructions.

Three experienced pathologists, blinded to the group allocation and transplantation outcome, assessed the biopsies with the Karpinski–Remuzzi score for chronic histopathological changes^8^, graded the acute tubular injury^9^ and assessed the expression (stain) intensity of PPARg in the glomeruli and in the tubules. The semi-quantitative score was 0 for absent expression, 1 for weak, 2 for moderate and 3 for strong.

Technical details can be found in the Supplementary Material.

### Statistical analysis

Clinical characteristics of donors and recipients were compared by unpaired T-test or ANOVA for continuous variables, while the discrete variables were analysed by X^2^-test.

A Pearson normality test was performed to assess whether the values of the protein quantification were normally distributed among the different groups. We used unpaired T-test or one-way ANOVA respectively for the analysis between two groups (AKI vs non-AKI and DCD vs DBD) or among four groups (G1, G2, G3 and G4) when the normality test was passed; Mann–Whitney or Kruskal–Wallis test was performed for comparison between two groups or among four groups when normal distribution was not achieved.

ANOVA analysis was also performed to assess the differences in the histological scores of chronic (Remuzzi–Karpinski score) and acute injury (ATI score) among the groups.

Spearman correlation analysis was performed between the detected protein quantity and the donor creatinine ratio (creatinine at retrieval/creatinine baseline) and between the PPARg intensity detected in the western blot analysis and the semiquantitative score assigned to PPARg expression in the immunohistochemistry analysis of both the tubular and the glomerular compartments.

A p-value of < 0.05 was considered significant. The statistical analysis was performed using GraphPad Prism 8.

## Results

### Selected population

**Figure 1:**
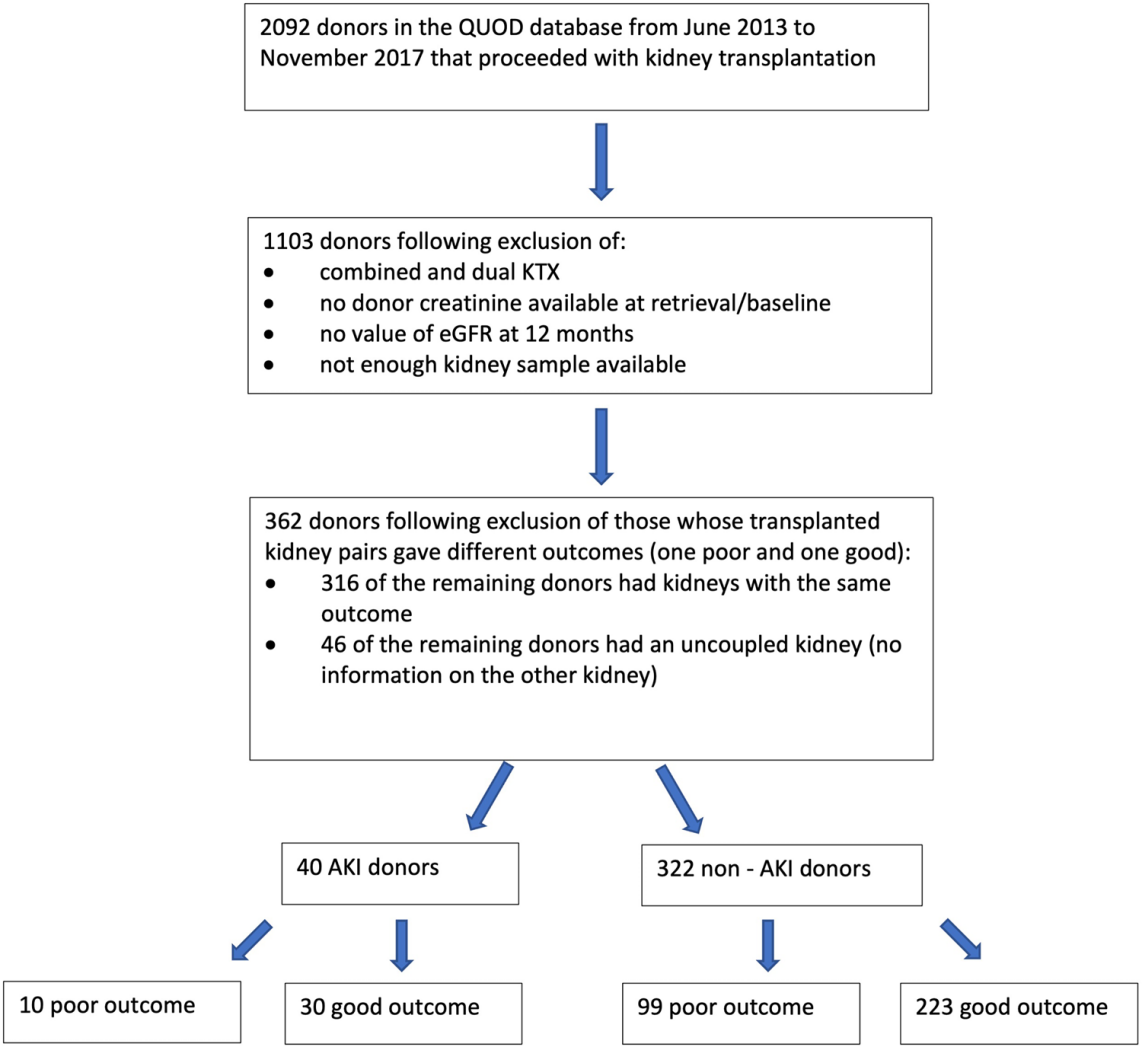
Flow diagram describing the process of sample selection.

We selected RNA-later samples and histology slides from 40 donors from the national QUOD biobank obtained from July 2014 to March 2018. In Fig. 1 a flow diagram represents the case numbers obtained throughout the process of sample selection.

After the exclusion criteria were applied, G1 became the reference group, with the lowest number of suitable samples, and therefore the other study groups (G2, G3 and G4) were chosen to match G1. One of the members of G3 was excluded due to the inadequacy of the related kidney biopsy. That group was therefore made of 9 samples.

We compared the clinical and demographic variables of donor, recipient and transplantation between the two donor categories (AKI and non-AKI) and no statistically significant differences were observed (Table S2). The distribution of variables across outcome groups (G1, G2, G3 and G4) and their comparisons are illustrated in Table 1. The analysis showed a lower donor mean age and KDPI for G2 compared to the other groups and the longest time spent on dialysis for G4.

**Table 1:** Donor and Recipient demographic and clinical variables for the selected sample cohort.

	G1 (n = 10)	G2 (n = 10)	G3 (n = 9)	G4 (n = 10)	p value
Kidney insult	AKI	AKI	No AKI	No AKI	
Post-transplant outcome	Poor	Good	Poor	Good	
Donor					
Age mean (± SD)	59.5 (± 7.89)	49.2 (± 9.64)	59.2 (± 8.14)	59.8 (± 8.48)	0.023^1^
Gender (M/F)	3/7	8/2	6/3	4/6	0.35
BMI mean (± SD)	26.5 (± 2.9)	28.1 (± 6.04)	25 (± 4.67)	26.6 (± 1.78)	0.47
Donor type (DBD/DCD)	7/3	7/3	6/3	10/0	0.67
KDRI mean (± SD)	2 (± 0.43)	1.45 (± 0.38)	1.76 (± 0.31)	1.62 (± 0.51)	0.016^2^
AKI type 1/2/3 (n)	7/3/0	6/1/2	–	–	
Transplantation					
CIT (min) mean (± SD)	871.4 (± 311.9)	743.1 (± 278.4)	1068 (± 474.7)	910.4 (± 439.6)	0.35
Recipient					
Age mean (± SD)	52.9 (± 15.78)	48.3 (± 14.83)	51.4 (± 12.95)	57.4 (± 14.73)	0.58
Gender (M/F)	5/5	6/4	8/1	6/4	0.78
BMI mean (± SD)	29.6 (± 4.4)	27.5 (± 5.2)	27.9 (± 5.1)	25.2 (± 5.4)	0.39
Re-transplantations (n of cases)	1	2	0	2	
Days on dialysis mean (± SD)	1116 (± 720)	1719 (± 1033)	656 (± 115)	1915 (± 2132)	0.043^1^
12mo eGFR mean (± SD)	25 (± 10)	68.3 (± 19.45)	32.6 (± 7.86)	66.2 (± 10.6)	0.043^3^

^1^Dunnett’s T3 multiple comparison across the groups did not show statistical difference.

^2^Dunnett’s T3 multiple comparison across groups showed a difference between G1 and G2 (p = 0.019).

^3^Dunnett’s T3 multiple comparison across groups showed a differences between: G1 and G2 (p = 0.0003), G1 and G4 (p < 0.0001), G2 and G3 (p = 0.013), G3 and G4 (p = 0.0031).

As per study design/outcome stratification, the eGFR at 12 months had lower values for the poor outcome groups (25 ml/min and 33 ml/min respectively for G1 and G3) compared to the good outcome ones (68 ml/min and 66 ml/min observed respectively in G2 and G4; p < 0.05 for all the relevant multiple comparisons across the groups).

### Molecular comparison between outcome groups

Each of the four groups consisted of n = 10 kidney biopsies from different donors, except for G3 (non-AKI—poor outcome), in which only 9 samples could be analysed.

The proteins related to mitochondrial biogenesis and dynamics (total DRP1, ratio PDRP1/DRP1, MFN1 and 2, PGC1α/β) did not show different expression patterns between the groups (data not shown). However, trends towards different levels of MFN1 (higher) and PGC1α/β (lower) were observed in the poor outcome groups (G1 and G3) compared to the good outcome ones (G2 and G4), irrespective of AKI status.

The markers of cellular stress γGT and HSP70 were consistent across the groups, and so were the pro-fibrotic markers TGFβ, HGF, PDGFR and IGBP7 (data not shown).

**Figure 2:**
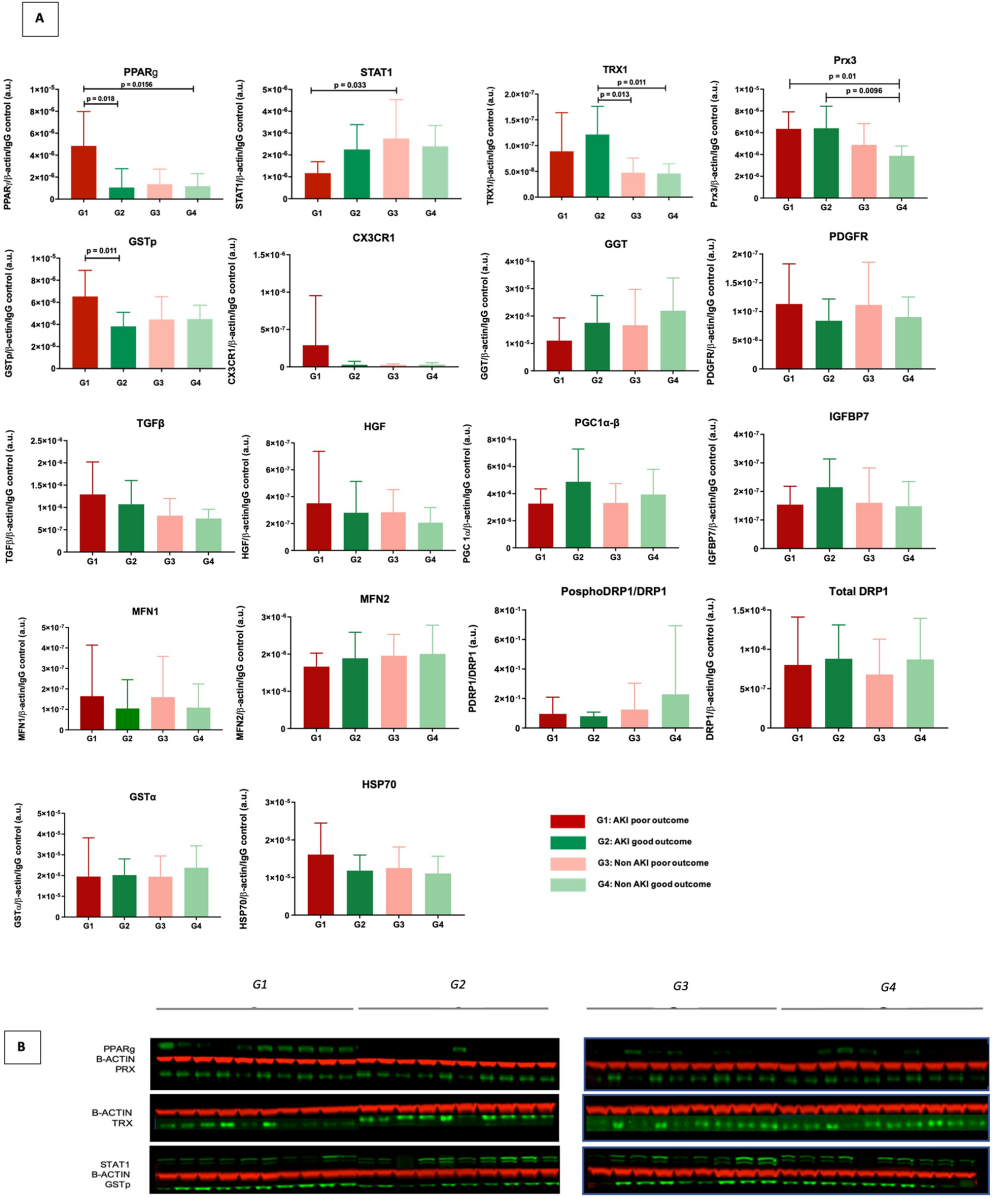
Histograms reporting expression intensities of western blot analysis of the proteins differently expressed in the four donor groups (**A**). Representative imaging of the immuno-blots for PPARg and PRX, TRX, STAT1 and GSTp in the four groups, with beta actin as loading control (**B**).

With regards to mitochondrial and cellular metabolism, PPARg was found to be nearly four-fold increased in G1 compared to all the other groups, with statistical significance reached versus G2 (p = 0.018) and G4 (p = 0.016) (Fig. 2).

Concerning inflammation, the behaviour of STAT1 and CX3CR1 was noteworthy. The levels of STAT1 were equal in the kidneys with good post-transplant function, without apparent impact of the ischemic injury (no difference due to presence of AKI). In contrast, the grafts which performed poorly after transplantation, showed dichotomous expressions of STAT1, being at its lowest in the AKI group (G1) and almost tripling in the non-AKI group (G3) (p = 0.033) (Fig. 2B). The chemoattractant molecule CX3CR1 was rather specifically detected only in G1 samples, with nearly absent signal in the other groups.

Finally, we found relevant variations in most of the proteins related to mechanisms of cellular protection. Both antioxidant enzymes TRX and PRX3 were increased in the AKI grafts versus the non-AKI counterparts (Fig. 2C,D), suggesting activation of the oxidative stress response. In addition, TRX expression was almost double in the AKI grafts which recovered successfully (G2), compared to non-AKI groups with poor (G3) and good (G4) outcomes (p values respectively 0.013 and 0.011). Opposite to these molecules, the detoxifying enzyme GSTp, was more expressed in G1 than in all other groups. However, statistical significance was reached only in the comparison with G2 (p = 0.011), where the expression of the protein nearly halved (Fig. 2E).

The correlation analyses between protein expression and the severity of kidney insult, donor age, and the comparison of molecular profiles of AKI vs non-AKI and of DCD vs DBD donors are described in the Supplementary Results.

### Histology

The histology was slightly impaired by some technical issues as no glomeruli were found in 10 biopsies; 3 in G1, 2 in G2, 3 in G3 and 2 in G4. These cases were not included.

**Figure 3:**
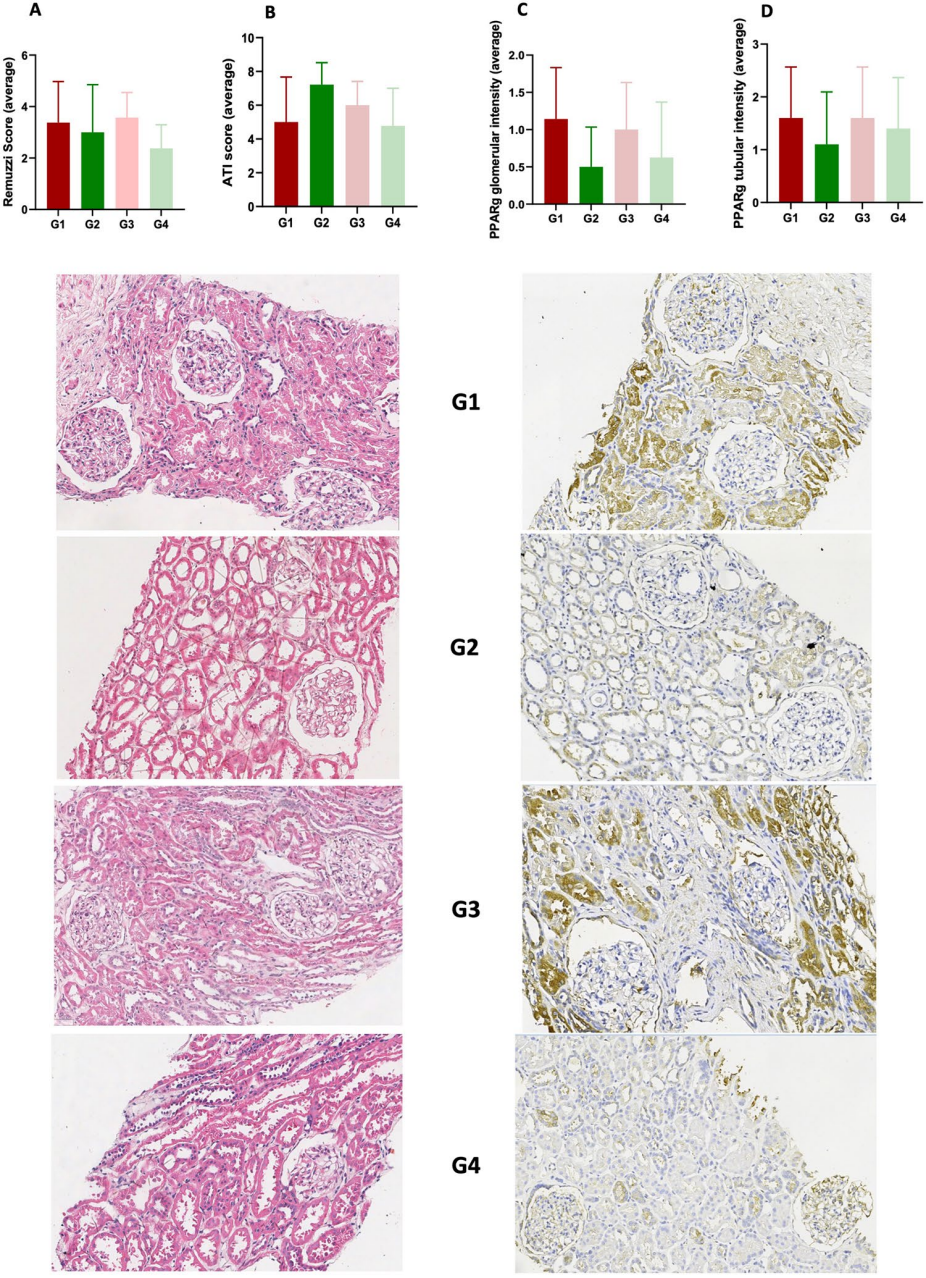
On the top of the figure, histograms show the average Remuzzi–Karpinski score (**A**), ATI score (**B**), intensity of PPARg expression in renal glomeruli (**C**) and tubules (**D**) across the study groups. On the lower part, representative images of histology (on the left) and immunohistochemistry (on the right) are shown across the study groups.

All the analysed slides showed a mild to moderate score of chronic injury, with a Remuzzi–Karpinski score ranging from 1 to 6. The mean score was 3 for both groups with AKI (± 1.6 SD for G1 and ± 1.85 SD for G2), while it was 4 (± 0.98 SD) for G3 and 2 (± 0.92 SD) for G4. No statistical significant difference was found between the groups (1-way ANOVA, p = 0.375) (Fig. 3A).

The acute tubular injury score ranged from 2 to 10 on a 0–16 scale. The average ATI score was 5 (± 2.68 SD) for G1, 7 (± 1.3 SD) for G2, 6 (± 1.41 SD) for G3 and 5 (± 2.22 SD) for G4. Although the 1-way ANOVA analysis did not show statistically significant differences among the groups (p = 0.059) we observed a trend for lower ATI scores in the group with AKI and poor outcome compared to the kidneys with AKI that had a favourable outcome (Fig. 3B).

Immunohistochemical analysis confirmed increased expression of PPARg in G1 both in the tubules and in the glomeruli, compared to the other groups. While the intensity of this marker was quite similar in the tubules across all the groups, the glomerular pattern showed some differences, although they did not reach statistical significance (Fig. 3C,D). In particular, we found greater glomerular intensity of PPARg in the groups G1 and G3, both characterized by poor outcome, compared to G2 and G4 which had lower PPARg expression and exhibited good outcomes. We then performed a correlation analysis between the PPARg signal in the Western Blot analysis and the immunohistochemical score of expression in the corresponding slides. We observed a direct positive correlation between the intensity of expression of PPARg in the immunohistochemistry and the Western Blot analysis (r = 0.425, CI 0.050–0.695; p = 0.024). This correlation was even stronger when looking specifically at the glomerular areas (glomerular immunohistochemical scores) (r = 0.473, CI 0.11–0.725; p = 0.011), while it was not present when analysing the tubules stain only (r = 0.136, CI − 0.197 to 0.441; p = 0.408).

## Discussion

The use of kidneys procured from deceased donors who have experienced AKI increases the risk of poorer long term graft function, especially when older donors and higher degrees of AKI are included^1,2^. In our study we aimed to define whether donor kidneys with AKI and poorer transplant outcomes present a distinguishable molecular profile and whether it is possible to identify potential molecular targets for reconditioning.

The role of peroxisome proliferator-activated receptors (PPARs) has been the target of much scientific interest in the last decade. In the kidney, the subtype PPARg is expressed in each renal area with different regulatory roles^10^. In the juxtaglomerular region, its activation regulates the vascular tone and blood pressure through an interaction with several components of the renin angiotensin system. The metabolism of fatty acid substrates and the activation of gluconeogenesis are controlled by PPARg in the proximal and distal tubules in the cortex of the kidney.

Research in rodent models has highlighted the role of PPARg in attenuating inflammatory and fibrotic processes also through the alternative macrophage activation, consequently, slowing the onset of chronic kidney failure^11,12^.

In our study we found significantly increased levels of PPARg in the group with acute kidney injury that had a poor post-transplant outcome. This observation may seem in contrast with the premises of the previously quoted studies. However, we suggest that the increased levels of PPARg that we observed may be part of a feedback response upon the ischemic insult. Although a response in terms of increased PPARg expression in the setting of ischemia/reperfusion injury has not been clearly described, an increased level of proteins and metabolites related to fatty acid metabolism was found in a rodent model of IRI, potentially as a response to oxidative phosphorylation dysfunction in the mitochondria^13^.

It is possible that only the severely injured kidneys were not able to meet the metabolic demands following the acute ischemic injury and this might have prompted a surge in the expression of PPARg to maximize its beneficial effect. In line with this observation, a recent article found increased expression of proteins involved in the PPAR signalling pathway in the perfusate of kidneys with suboptimal 1-year function^14^. In addition, in our study this pattern of PPARg expression was more evident in glomeruli than in tubular cells, as witnessed by the stronger correlation between the western blot expression and the immunohistochemical localization of PPARg. This evidence suggests that if a connection exists between PPARg expression and the regenerative capacity of an acutely injured kidney undergoing ischemia and reperfusion, this may be mediated by its biological effects on the juxtaglomerular cells. If further studies confirm this theory, an entire therapeutical field may open towards in-vivo or ex-vivo employment of pharmacological PPARg agonists, as thiazolidinediones, to prevent the detrimental pro-fibrotic effects of ischemia and reperfusion in renal grafts.

The involvement of antioxidant and detoxifying enzymes in the development of ischemia and reperfusion injury has been well established^15^.

There is growing evidence in the literature on the protective effect of the oxidative stress response and the importance of detoxifying agents in the prevention of chronic kidney disease after an acute ischemic insult^16,17^. Urinary thioredoxin 1 and glutathione S-transferase have been extensively studied as markers of acute kidney injury in the clinical setting^18,19^, while peroxiredoxin 3 was shown to be overexpressed in kidneys with acute tubular necrosis, particularly in the organs that recovered well after acute injury, suggesting a potential role as biomarker^17^.

In our study, we found enhanced levels of PRX3 and TRX1 in the acutely injured kidneys, as expected. In particular, the levels of TRX1 among the AKI samples were higher in the organs that had a good post-transplantation outcome. PRX3 and TRX1 are antioxidant enzymes both downstream targets/products of nuclear factor erythroid-derived 2-like 2 (NRF2)^20^. It could be hypothesized that, upon an acute insult, the cellular redox mechanisms are boosted at the transcriptional level to overcome the accumulation of oxidation products, and only the kidneys that have a prompter response of these mechanisms can more successfully recover renal function.

Conversely the detoxifying agent GSTp was only increased in the AKI group that had a poor outcome. This is in line with previously published literature where increased levels of GSTp were found in the urine of patients developing severe AKI upon admission to critical care units or after cardiac surgery^19,21,22^ and in patients with chronic kidney conditions^23^. GSTp is generally expressed in the distal tubules, so our findings may reflect the importance of the localization of the ischaemic insult in the context of recovery.

A few animal and human studies have focused on the role of the Janus kinase/signal transducer and activator of transcription (JAK/STAT) pathway in the pathogenesis of I/R injury. Some studies have shown that preventing JAK/STAT phosphorylation and therefore its activation, is effective in slowing the progression of I/R injury in rodent models^24,25^. A more recent study has shown that the role of JAK/STAT activation is more complex in the development of the acute injury and of potential detrimental long-term consequences on the renal function^26^. Kemmner et al. have performed experiments of acute renal ischemic injury on STAT1 knockout mice. The authors showed that, despite a mitigation of tubular necrosis and inflammatory infiltration, the knockout mice developed more fibrosis from the ischemic insult compared to the wild-type. They also found that the macrophages infiltrating the kidneys of the STAT−/− mice were of the alternatively activated phenotype, which may suggest that STAT1 deficiency drives the shift of macrophages towards this proinflammatory transformation. A similar modulating role on innate immune response has been found at the post-transcriptional level in the STAT1 pathway^27^. In our study we found the lowest level of STAT1 in the samples from the group with AKI which developed a poor outcome. Differently from the previously cited papers, we show that STAT1 has an opposite behaviour depending on the presence or not of AKI before the retrieval. The levels of STAT1 were not low in all the poor outcome groups, but only when an acute ischemic injury had occurred beforehand, suggesting these kidneys might be more at risk of developing a pro-fibrotic profile as described in the knock-out models.

A similar pattern was observed in the histological assessment of acute tubular injury. AKI kidneys which had an unfavourable outcome at 1 year tended towards a lower ATI histological score while, surprisingly, a more severe histological pattern of acute injury was seen in kidneys that resulted in a better function.

These results suggest that when an ischemic event adds to a condition of STAT1 under-expression a pro-fibrotic response ensues more rapidly, determining poorer outcomes post-transplantation.

The retrospective nature of the study implies a certain degree of selection bias which is reflected in the distribution of different degrees of AKI in the groups, uneven numbers of DCD/DBD and a lower median donor age in the G2 group. However, our analysis showed comparable molecular expression between DCD and DBD within the AKI group, suggesting that the role of AKI on the studied molecular markers of damage was not predominantly influenced by the mechanism of death, and the donor age was not correlated to the protein expression patterns observed in the samples either.

We acknowledge that the eGFR at 1-year post-transplant may seem a weak indicator of long-term outcome. However, evidence is emerging on the role of eGFR in the first-year post-transplantation as predictor of the transplantation outcome^7^. Moreover, we selected the samples to maximize the difference of eGFR between the groups.

Due to the limited numbers of a severe form of AKI in transplanted kidneys it was not possible in this study to detect significant differences among the various AKI degrees. More donor kidneys with severe AKI are required to evaluate potentially different molecular profiles versus less severe AKI. We wonder whether this accrual will be practically possible as we have used the UK QUOD biobank which is the largest bioresource of its kind and includes kidney biopsies obtained from more than 7000 deceased donors. Since recipient-related factors have a noticeable impact on the transplantation outcome, we have attempted to minimize this ‘recipient’ effect by matching the most important recipient variables among the groups. In addition, we excluded from our study donors whose kidneys were transplanted and had different clinical outcomes in the recipients, as it is unlikely that two kidneys from the same donor may have very different post-transplantation outcomes aside from factors related to the recipients and technical/surgical aspects.

Summarizing, in this study we found a specific molecular pattern in kidneys retrieved from donors with AKI that proceed towards worse function after transplantation. The increase of PPARg in this category of organs suggests a role of lipidic metabolic dysfunction in the development of kidney failure after a severe ischemic damage. Conversely, a reduced expression of STAT1 has a detrimental effect specifically when additional to a pre-retrieval acute ischemic injury. If these findings will be supported by further research, these molecular pathways could become a crucial therapeutic target for the pre-transplant reconditioning of severely injured kidneys.

### Supplementary Information

Supplementary Information 1.

Supplementary Information 2.

## Data Availability

All data analysed and reported in this manuscript are availale to consultation upon request addressed to the author Flavia Neri (mailing address: Hospital Papa Giovanni XXIII, General Surgery 3, square OMS 1, 24127 Bergamo, Italy e-mail: flavia.neri84@gmail.com).
