# Supplementary material for: Renal biopsies from donors with acute kidney injury show different molecular patterns according to the post-transplant function
Source: Sci Rep. 2024 Mar 19;14:6643. doi: 10.1038/s41598-024-56277-x (PMC10951245; doi:10.1038/s41598-024-56277-x)
Supplement: Supplementary file 1 — Supplementary Information 1. [file 41598_2024_56277_MOESM1_ESM.docx]

SUPPLEMENTAL DIGITAL CONTENT

**Materials and methods**

*Tissue lysis and protein concentration determination*

The cortical kidney biopsies were lysed in 300 μl RIPA buffer per 10 mg of tissue. The RIPA buffer contained a protease (Roche, USA) and phosphatase (Sigma, UK) inhibitor cocktail. Homogenization was obtained through at least 3 cycles (6000 Hz, 30 seconds) on a beads beater (Precellys homogenizer, Bertin technologies, France), separated by 1 minute of cooling on ice. The supernatant was collected and the concentration of the proteins in the samples was assessed by BCA assay (Thermo Fisher Scientific, USA) according to manufacturer’s instruction.

*Immunoblotting*

Selected proteins were analysed by immunoblotting. According to the assessed protein concentration, each sample was either diluted or concentrated (Amicon® Ultra-0.5 mL Centrifugal filters) to the same starting total protein concentration (1 μg/μl) . Then samples were diluted 1:1 in Laemmli buffer and denaturated at either 95°C for 5 minutes (for antibody panels 2, 3 and 4) or at 60°C for 10 minutes (for panel 5). Ten µg of protein per sample were then loaded onto either 4-12% (panel 2, 4 and 5) or 12% (panel 3) pre-cast SDS-PAGE gels (Bio-Rad, USA) (Table S1). On each gel 20 samples (two groups) run alongside a molecular weight marker and an internal positive control (0.04 μg normal rabbit IgG (sc-2027, Santa Cruz)) to allow for signal normalisation and comparison across different gel runs.

After completion of electrophoresis (140 V for 90 minutes), the proteins separated by SDS-PAGE were transferred to hydrophobic PVDF membranes (Merck Millipore, USA) in transfer buffer (25 mM Tris, 192 mM glycine and 10% methanol) at 4°C for 2 hours. The PVDF membranes were then blocked for 1 hour at 4°C in TBS buffer (25 mM Tris, pH 7.5, 0.15 M NaCl) containing either 5% milk (for panels 2, 3 and 4) or 5% BSA (panel 5). The membranes were then incubated overnight at 4°C with the primary antibodies diluted in TBST buffer (25 mM Tris, pH 7.5, 0.15 M NaCl, 0.05% Tween 20) containing either 5% milk (for panels 2,3 and 4) or 5% BSA (panel 5), at the dilutions illustrated in Table S1. Beta-actin served as loading control for each sample. Membranes were washed for 30 minutes with 5 changes of TBST buffer and then incubated with the secondary antibodies (IRDye 680RD-conjugated anti-mouse and IRDye 800CW-conjugated anti-rabbit, 1:15,000, Li-Cor) at room temperature, for 1h protected from light. Membranes were then imaged in an Odyssey CLx system (Li-Cor Nebraska, USA). The detected signal was quantified and normalized to β-actin on the same blot, and to the positive IgG rabbit control to allow for inter-gel comparisons.

All the membranes underwent a process of antibody stripping, through incubation with acid (pH 2) mild stripping buffer (25 mM Glycine-HCl, 1% SDS) at room temperature for 1 hour. After washing the membranes with TBS and TBST buffer for 30 minutes, they underwent blocking and overnight incubation with an additional set of primary antibodies at 4°C (as detailed in Table S1). The membranes were then incubated with the secondary antibodies and imaged as described above.

Table S1: Panels of primary antibodies used in the Western Blot analysis and relative concentrations

| **SDS-PAGE gel type** | 4-12% | 12% | 4-12% | 4-12% |
| --- | --- | --- | --- | --- |
| **PANEL** | 1 | 2 | 3 | 4 |
| **PRIMARY ANTIBODIES (CONCENTRATION)** | PGC1α (1:2,000) | Hsp70 (1:1,000) | PDGFRα (1:1,000) | P-DRP1 (1:1,000) |
|  | MFN 2 (1:4,000) | GSTa (1:1,000) | HGF (1:250) | γGT (1:5,000) |
|  | PPARγ (1:2,000) | TRX1 (1:25,000) | CX3CR1 (1:5,000) | TGFβ (1:1,000) |
|  | PRDX (1:50,000) | βACTIN (1:16,000) |  | GSTp (1:5,000) |
|  | βACTIN (1:16,000) |  |  |  |
| AFTER STRIPPING OF THE ANTIBODY FROM THE MEMBRANES | | | | |
| **PANEL** | 1a | 2a | 3a | 4a |
| **PRIMARY ANTIBODIES (CONCENTRATION)** | MFN1 (1:10,000) | STAT1 (1:10,000) | βACTIN (1:16,000) | DRP1 (1:2,000) |
|  | βACTIN (1:16,000) | βACTIN (1:16,000) |  | IGFBP (1:1,000) |
|  |  |  |  | βACTIN (1:16,000) |

**Abcam, UK**: PGC1α+β (ab188102), βactin (ab6276), MFN2 (ab205236), MFN1 (ab129154), PPARγ (ab178860), Hsp70 (ab181606), HGF (ab178395), GSTα (ab207413), TGFβ (ab179695), TRX (ab133524), PRDX (ab128953), STAT1 (ab109320), DRP1 (ab184247), PDGFRα (ab203491), CX3CR1 (ab184678), γGT (ab109427), IGFBP (ab171085), GSTπ (ab138491)

**Cell Signaling, US**: P-DRP1 (#4867)

*Immunohistochemistry*

Immunohistochemistry was performed on 4μm thick tissue sections. Slides were deparaffinized using xylene followed by rehydration in different concentrations of ethanol. Epitope retrieval was achieved through heating of the tissue in citrate buffer, and it was followed by overnight incubation with the primary antibody. Antibody against peroxisome proliferator activated receptor PPARg (AHP1461, AbdSerotec, Puchheim, Germany) was diluted to 1:1000 in 1% bovine serum albumin. Envision detection system (Dako, Amsterdam, The Netherlands) was used for detection. Samples were stained with DAB (Dako, Amsterdam, The Netherlands), and counterstained with Mayer’s hematoxylin (Merck Millipore, the Netherlands). For quantification purposes one full length section (typically 10-15 mm) per sample was quantified. Stained slides were scanned at 400X magnification using Philips’ IntelliSite Ultra Fast Scanner (Philips, Eindhoven, the Netherlands).

**Results**

Table S2: Donor and Recipient demographic and clinical variables compared between AKI and non-AKI samples

| **Kidney insult** | | **AKI** | **no AKI** | **p value** |
| --- | --- | --- | --- | --- |
| DONOR | age mean (± SD) | 54.4 (± 10.07) | 59.53 (± 8.09) | 0.089 |
|  | DCD (n) | 6 | 3 | 0.501^1^ |
|  | KDRI mean (± SD) | 1.74 (± 0.49) | 1.69 (± 0.42) | 0.722 |
| TRANSPLANTATION | CIT (min) mean (± SD) | 807.3 (± 295.2) | 985.2 (± 450.9) | 0.157 |
| RECIPIENT | age mean (± SD) | 50.6 (± 15.09) | 54.6 (± 13.87) | 0.396 |
|  | BMI mean (± SD) | 27.3 (± 4.7) | 25.8 (± 3.45) | 0.38 |
|  | Re-transplantations (n of cases) | 3 | 2 | 0.951^1^ |
|  | Days on dialysis mean (± SD) | 1435 (± 926) | 1443 (± 1739) | 0.549 |
|  | 12mo eGFR  mean (± SD) | 46.6 (± 26.8) | 50.26 (± 19.53) | 0.632 |

^1^ Yates correction was applied to the Chi square calculation

*Correlation analysis of protein expression with donor creatinine retrieval/baseline ratio - recipient eGFR and donor age*

We further explored whether a correlation existed between protein expression and the severity of kidney insult, expressed as a continuous variable as the ratio between the donor creatinine at retrieval and the baseline. We found a significant, although weak, negative correlation for STAT1 (r = -0.342; p = 0.033) and positive correlations for TRX (r = 0.39; p = 0.015), PRX3 (r = 0.53; p = 0.0006) and TGFβ (r = 0.37; p = 0.02).

When analysing the correlation between protein expression and 12-month eGFR we found a positive correlation for MFN1 (r = 0.355; p = 0.026) while it was negative for PPARg (r = -0.49; p = 0.0015) and GSTp (r = -0.332; p = 0.039).

We also conducted a Spearman's correlation analysis between protein expression and donor age, in order to assess whether the younger age of G2 was influencing the different molecular patterns observed in our previous results. None of the investigated proteins showed a significant correlation with the donor age.

*Comparison of molecular profiles of AKI vs non-AKI samples*

With the intention of describing the molecular pattern of damage in acutely injured kidneys offered for transplantation we analysed the expression of proteins involved in AKI (listed in the materials and methods section) through Western Blotting.

The immunoblotting analysis showed comparable expression of most of the investigated proteins between the AKI and non-AKI donor samples except for the pro-inflammatory STAT1 that was decreased in the AKI group (p = 0.033), and the antioxidant enzymes TRX and PRX3 which showed a two-fold (p = 0.001) and nearly a 50% (p = 0.005) increase in the AKI group respectively (data not shown). The trend of two proteins, PPARg and CX3CR1, was noteworthy: despite a lack of statistical significance, their mean levels in the AKI samples were almost twice as much as in the non-AKI group.

*DCD vs DBD within the AKI group*

Aware of the impact of the type of death on the acute ischaemic injury of the kidney, we compared the results of the immunoblotting analysis between the samples from DCD and DBD donors within the AKI group. The expression of all proteins was comparable in the two subcategories of donors (DBD and DCD) that had experienced AKI (data not shown).

A fair comparison between DCD and DBD samples in the non-AKI group was not possible, as the DCD cases were too few (only 3 DCD versus 16 DBD) and all of them were unequally represented in the poor outcome group.
